# A descriptive pilot study of cytokine production following stimulation of ex-vivo whole blood with commercial therapeutic feline hydrolyzed diets in individual healthy immunotolerant cats

**DOI:** 10.1186/s12917-017-1219-9

**Published:** 2017-10-06

**Authors:** Aarti Kathrani, Jennifer A. Larsen, Gino Cortopassi, Sandipan Datta, Andrea J. Fascetti

**Affiliations:** 1 Veterinary Medical Teaching Hospital, School of Veterinary Medicine, University of California-Davis, Davis, CA 95616 USA; 2 Department of Molecular Biosciences, School of Veterinary Medicine, University of California-Davis, Davis, CA 95616 USA; 3 Present address: School of Veterinary Sciences, University of Bristol, Langford House, Langford, Bristol, BS40 5DU UK

**Keywords:** Feline, Hydrolyzed diet, Cytokine, Ex-vivo

## Abstract

**Background:**

Hydrolyzed diets are used in companion animals for the diagnosis and treatment of adverse food reaction. Similarly, hydrolyzed formulas are used in human infants with severe inflammatory bowel disease or milk allergy, and these must meet the standard of hypoallergenicity through rigorous testing. Unfortunately, no standards are currently applied to hydrolyzed veterinary therapeutic diets, and data for the immunogenicity of feline diets is also not available. Therefore, the main aim of this pilot study was to determine if ex-vivo whole blood stimulation assays could be used to characterize the cytokine response to hydrolyzed commercial diets in a small number of individual healthy immunotolerant cats. This approach has also been used to investigate cytokine production in response to cow milk protein in humans and currently similar studies do not exist in companion animals. Nine healthy cats previously eating the same basal diet were divided into groups and fed one of three hydrolyzed diets exclusively for 6 weeks. Heparinized whole blood was collected from each cat before and after the feeding trial. Ex-vivo whole blood stimulation assays were performed using crude extracts of the basal diet as a positive control, as this diet contained the same proteins present in the hydrolyzed diet but were intact, saline as a negative control, and each cat’s respective hydrolyzed diet. Supernatants were collected and analyzed for tumor necrosis factor-alpha, interleukin-10 (IL-10), and interleukin-4 using enzyme-linked immunosorbant assay.

**Results:**

Seven cats produced detectable amounts of the anti-inflammatory cytokine IL-10 upon stimulation with the basal diet. Two cats produced detectable amounts of IL-10 upon stimulation with a hydrolyzed soy-based diet and one cat produced a detectable amount of IL-10 upon stimulation with a hydrolyzed chicken-based diet (>125 pg/mL).

**Conclusions:**

Results from this pilot study suggest that in some healthy immunotolerant cats, some hydrolyzed diets may elicit a similar cytokine response compared to their basal diet, which contained the same proteins intact. Therefore, animals may be able to recognize and react to some hydrolyzed forms of tolerated proteins, and may also suggest IL-10 as a target for investigation as a potential marker for dietary tolerance in cats, however further studies would be necessary to corroborate this. Further studies are also needed to determine if this would also be the same in immunologically naïve, sensitized and clinically hypersensitized cats.

## Background

Hydrolyzed diets contain peptides created from chemically or enzymatically treated proteins; these peptides are small enough to theoretically avoid a type 1 hypersensitivity immune response, by preventing cross-linking of two immunoglobulin E antibody receptors on a mast cell. A peptide of 10 kDa or less is considered adequate to avoid this response; however there is evidence to suggest that the size needed may actually be smaller than three to five kDa [1]. Hydrolyzed diets are used in companion animals to diagnose and treat cutaneous and gastrointestinal adverse food reactions (AFR) [2]. Likewise, hydrolyzed baby formulas are used commonly in infants with severe inflammatory bowel disease or cow milk allergy [3]. These formulas must meet the standard of hypoallergenicity through testing using in vitro and in vivo animal models as well as clinical assessment [4, 5]. As similar standards are not currently applied to hydrolyzed pet foods, the proteins in these diets may still retain their antigenic potential. This was demonstrated in one study, which indicated that a significant proportion (21%) of dogs sensitized to the intact protein still reacted adversely to the hydrolyzed diet [6]. Therefore, the most significant clinical problem with veterinary hydrolyzed diets may be the retention of antigenicity leading to continued clinical signs. Consequently, studies are needed to verify the immunological potential of these diets.

The current pilot study aimed to describe the immunological effects of 3 available feline hydrolyzed dry diets in individual healthy immunotolerant cats before and 6-weeks after consuming a hydrolyzed diet compared to a basal diet containing these proteins intact, by measuring cytokine production using an ex-vivo whole blood stimulation assay. This assay has been used to investigate cytokine release in response to various antigens such as cow milk protein in humans [7]. Although this ex-vivo protocol has been described in dogs using bacterial ligands as a stimulant [8–10]; to the authors’ knowledge this is the first pilot study to use commercial pet foods as a stimulant in companion animal studies. We assessed 3 cytokines known to be commonly modified in adverse immunological responses to food. An increase in the Th2 cytokine, IL4 and a decrease in the Th2 cytokine, IL10 have been shown to play an important role in the pathogenesis of food-induced gastrointestinal disorders in humans and mice [11–13]. Similarly, an increase in the Th1 cytokine TNF-alpha plays a role in cow-milk allergy in children and experimental food allergy in mice [14, 15]. The results of this study will help to describe the immunological response to hydrolyzed therapeutic diets in individual healthy immunotolerant adult cats; the results can be subsequently compared to immunologically naïve, sensitized and clinically hypersensitized cats in order to help characterize the pathogenesis of feline AFR and the role of hydrolyzed diets in the treatment of this disease.

## Methods

### Cats

Nine healthy intact female, specific pathogen free cats, residing in an existing colony at the University of California, Davis were used in this study. No abnormalities were noted on the basis of pre-study physical examinations. Mean age was 1.3 years (range 1 to 1.5 years), mean body weight was 3.6 kg (range 3.0 to 4.4 kg), and mean body condition score was 5/9 [16] (range 4/9 to 6/9). A standardized complete physical examination was completed on all cats prior to study initiation. All study cats were born to queens consuming the same commercial feline dry diet throughout gestation and lactation suitable for all life stages based on feeding trials.^1^ Following weaning, study cats were then fed the same dry diet suitable for maintenance^2^ (basal diet) until study initiation. This basal diet contained the same proteins present in the three hydrolyzed diets used in this study but intact.

Cats were group-housed by dietary treatment and received daily attention through activities such as petting and brushing, as well as access to numerous toys and scratching posts for behavioral enrichment. The facility maintains room temperatures between 18 and 24 **°**C, and has a 14 h light/10 h dark cycle. This experimental protocol was reviewed and approved by the Institutional Animal Care and Use Committee (IACUC) of the University of California, Davis (USA) (Animal Welfare Assurance Number A3433–01).

### Study protocol

The cats were allocated to three groups (*n* = 3) using a random number generator and were housed by dietary treatment throughout the study. Cats were adapted to their respective dietary treatment group while continuing to be fed the basal diet for 7 days prior to the start of the study (day −7 to 0). All cats had free access to food and water throughout the study.

At day 0, the basal diet was discontinued and three cats received a hydrolyzed chicken based diet^3^ (hydroC group), three cats received a hydrolyzed soy and chicken based diet^4^ (hydroSC group), and the remaining three cats received a hydrolyzed soy based diet^5^ (hydroS group). The hydrolyzed test diets were fed exclusively for 6 weeks. This duration was based on resolution of clinical signs in dogs in this time period when treated for food responsive enteropathy with a hydrolyzed diet [17]. Body weights and body condition scores were assessed by the same evaluator (AK) weekly and stool quality was monitored daily. On days 0 and 43 (at the start of the study and after the 6-week study period), six milliliters of blood were collected from each cat via jugular venipuncture into heparinized tubes following a 12 hour fast.

### Preparation of diets for use in ex-vivo whole blood stimulation assays

Ten grams of each dry food (the basal diet and each of the 3 hydrolyzed diets)^2,3,4,5^ collected from a new unopened bag was ground with sterile water using a clean pestle and mortar. The mixture was then transferred to a 50 ml sterile tube^6^ and incubated overnight at 4-degrees on a rotator. The tube was centrifuged at 8000 rpm for 1 minute at room temperature and the supernatant was filtered using a 0.22-μm polyethersulfone sterile filter^7^ in a sterile tissue culture hood.^8^ The protein content in each of the filtrates was determined using the Bradford assay.^9^ Working solutions of 500 μg/mL and 50 μg/mL were prepared for each of the dietary filtrates using phosphate buffered saline.^10^ These concentrations were chosen based on results of studies using lymphocyte proliferative assays to various milk allergens [18, 19].

### Blood analysis

Fresh heparinized whole blood, collected within 1 h before analysis, was mixed with 18 ml of RPMI^11^ containing penicillin/streptomycin^l^ and 1 ml plated in a 24 well plate.^12^ A negative control consisting of 111 uL of phosphate buffered saline^10^ and a positive control of 111 uL of 50 μg/mL and 500 μg/mL of basal diet and 111 uL of 50 μg/mL and 500 μg/mL of the respective hydrolyzed diet consumed by that cat during the 6 week study period were added to each well. All samples were analyzed in triplicates for a total of 18 wells. All plates were incubated at 37 degrees centigrade at 5% carbon dioxide for 24 h.^13^ The plates were spun at 2000 x g for 4 min and the supernatant harvested and stored at −80 **°**C until analyzed.

The Feline TNF-alpha DuoSet ELISA,^14^ Feline IL-10 DuoSet ELISA,^15^ and Feline IL-4 DuoSet ELISA^16^ were used to measure TNF-alpha, IL-4, and IL-10 production in the supernatants according to the manufacturers’ instructions. The triplicate cell culture wells were assayed individually. The optical density was measured at 450 nm with a 540 nm wavelength correction using a Bio-Tek Synergy H1 Multi-Mode,^17^ Absorbance Reader within 30 min and the data were analyzed using Gen5 Microplate Reader and Imager Software.^18^ All assays were performed in duplicate. The lower limit of detection of the assays was 15.6 pg/mL for TNF-alpha, 125 pg/mL for IL-10 and 62.5 pg/mL for IL-4.

### Data analysis

The mean of each of the duplicate cytokine concentrations was calculated. The mean of these values from the triplicate wells in the ex-vivo whole blood stimulation assay was then calculated for each of the diets and standardized to phosphate buffered saline by subtraction.

Differences in age, body weight, and body condition score between the three groups at inclusion was analyzed using the Mann-Whitney U test. A Wilcoxon signed-rank test was used to compare IL-10 concentrations following stimulation with the basal diet before and after the 6-week diet trial. Unfortunately, due to the small number of cats consuming each of the three hydrolyzed commercial diets, statistical analysis on cytokine production could not be performed among groups. Analyses were performed using IBM SPSS Statistics Version 23. Significance was defined as *P* ≤ 0.05.

## Results

### Cats

There were no significant differences in age, body weight, or body condition score between the three groups (*P* > 0.05). All cats remained weight stable and maintained normal stool quality throughout the 6 week study period. All cats completed the study.

### Ex-vivo whole blood stimulation assays

Numbers of cats that produced detectable amounts of IL-10, IL-4 and TNF-alpha following stimulation with 50 μg/mL or 500 μg/mL of the basal diet and the respective hydrolyzed diet before and after 6 weeks of consumption of hydrolyzed diets are presented in Table 1.

**Table 1:** Number of cats producing detectable concentrations of cytokines in ex-vivo whole blood stimulation assays

Cytokine	Timepoint	Basal diet		Respective hydrolyzed diet	
		50 μg/mL	500 μg/mL	50 μg/mL	500 μg/mL
IL-10	Day 0	0/9	5/9	0/9	2/9
	Day 43	0/9	7/9	0/9	3/9
IL-4	Day 0	0/9	0/9	0/9	0/9
	Day 43	0/9	0/9	0/9	0/9
TNF-alpha	Day 0	0/9	0/9	0/9	0/9
	Day 43	2/9	0/9	0/9	0/9

Number of cat blood samples producing detectable concentrations of interleukin-10 (IL-10), interleukin-4 (IL-4), and tumor necrosis factor-alpha (TNF-alpha) following ex-vivo whole blood stimulation assay with 50 μg/mL or 500 μg/mL of pre-study basal diet and the respective hydrolyzed diet, before and after a 6-week feeding trial of one of 3 hydrolyzed diets (*n* = 3 cats per group). No samples produced detectable cytokines following stimulation with the negative saline control

### Stimulation with basal diet

**Fig. 1:**
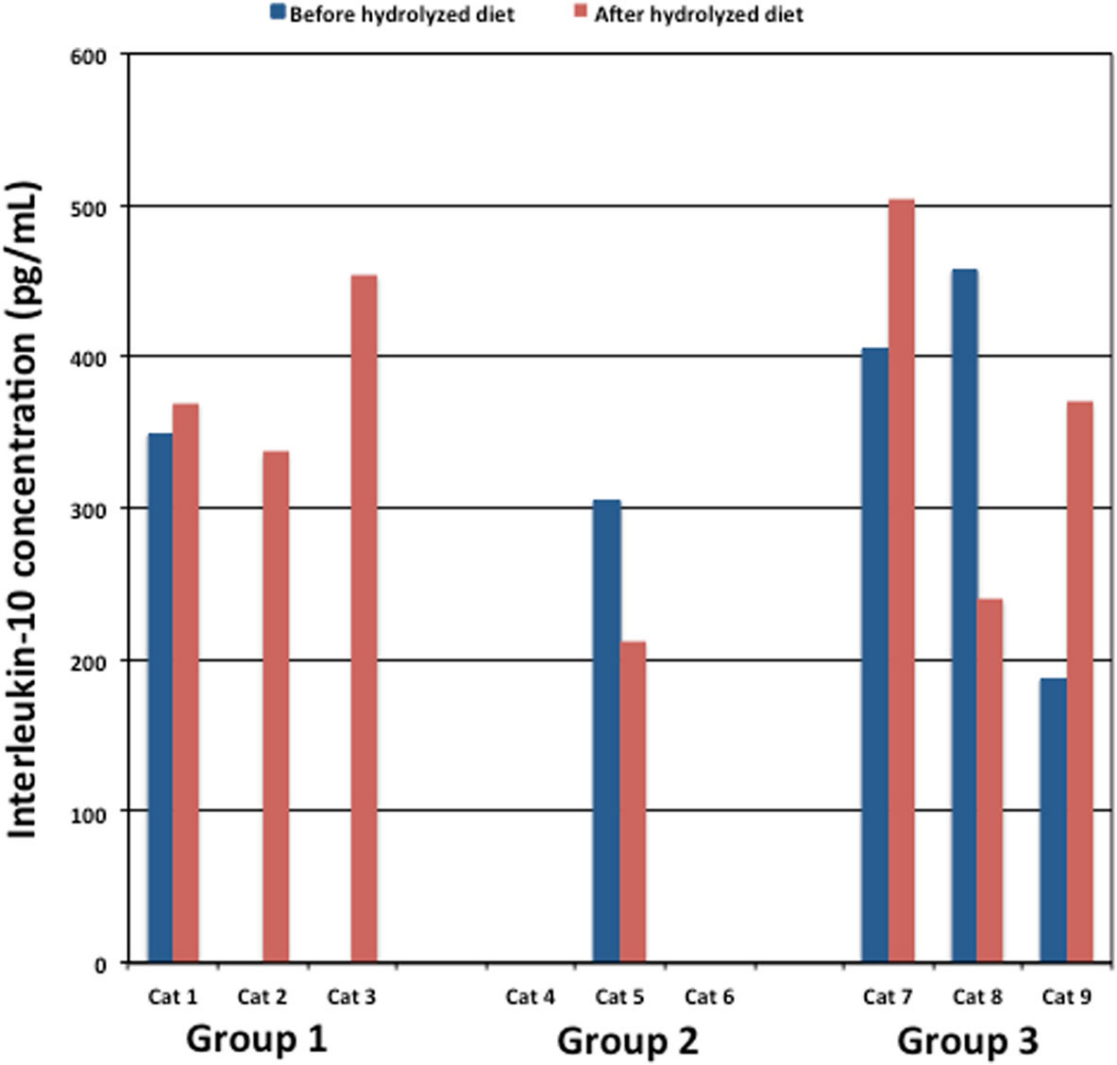
Comparison of interleukin-10 in supernatants from ex-vivo whole blood stimulation assays using the basal diet. Bar graph of interleukin-10 concentration in supernatant following ex-vivo whole blood stimulation assay for all cats using 500 μg/mL of Purina Cat Chow Complete Formula dry food, before and after 6 weeks of consuming the respective hydrolyzed diet (group 1 – hydroC, group 2 – hydroSC, and group 3 – hydroS)

On day 0 prior to starting the hydrolyzed diet, 5/9 cats produced detectable amounts of IL-10 on stimulation with 500 μg/mL of the basal diet (Fig. 1: mean 340.8 pg/mL, range 187.5–457.2 pg/mL). This number increased to 7/9 cats after consuming their assigned hydrolyzed diet for 6 weeks (Fig. 1: mean 355.6 pg/mL, range 212.7–503.7 pg/mL). Although two of the seven cats had lower IL-10 concentrations after 6 weeks compared to baseline (Fig. 1), there was no significant difference in IL-10 concentrations before and after 6 weeks (*P* > 0.05). No cat had detectable IL-10 concentration at baseline that was then undetectable at 6 weeks (Fig. 1). The two remaining cats that did not produce detectable levels of IL-10 following consumption of their respective hydrolyzed diet (hydroSC) produced TNF-alpha when stimulated with 50 μg/mL of the basal diet (65.8 pg/mL and 34.4 pg/mL respectively), but not on stimulation with 500 μg/mL of the basal diet.

### Stimulation with hydrolyzed diets

**Fig. 2:**
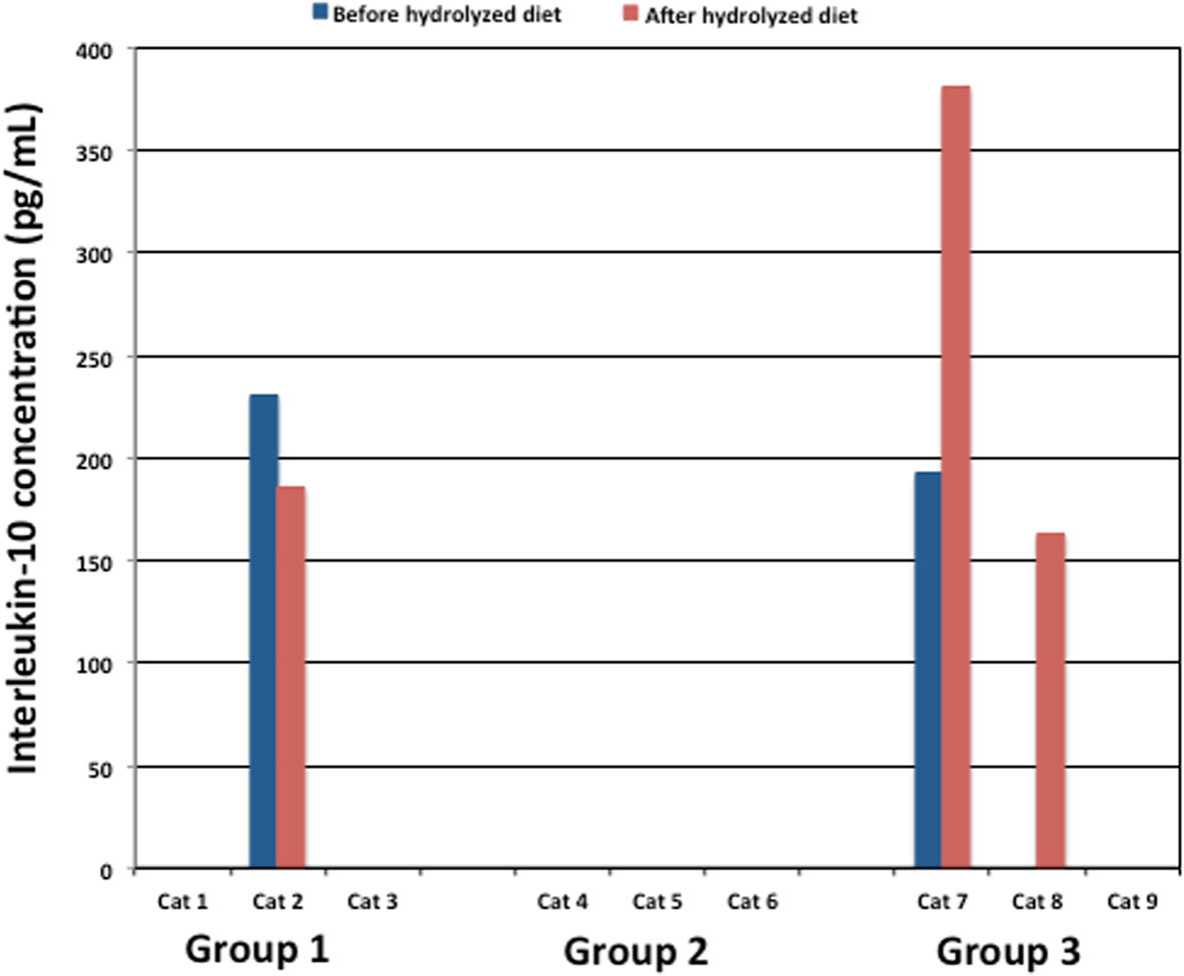
Comparison of interleukin-10 in supernatants from ex-vivo whole blood stimulation assays using hydrolyzed dry diets. Bar graph of interleukin-10 concentration in supernatant following ex-vivo whole blood stimulation assay for all cats using 500 μg/mL of hydrolyzed diet, before and after 6 weeks of consuming the respective hydrolyzed diet (group 1 – hydroC, group 2 – hydroSC and group 3 – hydroS)

One of the three cats in the hydroC group produced a detectable amount of IL-10 when stimulated with 500 μg/mL of the hydrolyzed chicken based diet both prior to and after 6 weeks of consumption (Fig. 2: 230.5 pg/mL and 185.5 pg/mL respectively).

None of the three cats in the hydroSC group produced detectable amounts of IL-10 following stimulation with the hydrolyzed soy and chicken based diet prior to or after 6 weeks of consumption (Fig. 2; <125 pg/mL).

One of the three cats in the hydroS group produced a detectable amount of IL-10 when stimulated with 500 μg/mL of the hydrolyzed soy based diet, prior to starting the diet trial (Fig. 2: 192.2 pg/mL). This same cat and a second cat produced detectable amounts of IL-10 when stimulated with 500 μg/mL of the hydrolyzed soy based diet after 6 weeks of consumption (Fig. 2: 380.5 pg/mL and 162.9 pg/mL).

Prior to starting and after 6 weeks of consumption of their assigned hydrolyzed dietary treatment, no cats in any group produced detectable amounts of TNF-alpha or IL-4 following stimulation with their respective hydrolyzed diet (<15.6 pg/mL and <62.5 pg/mL, respectively).

## Discussion

In this pilot study, an ex-vivo whole blood stimulation assay was used to describe cytokine production following stimulation with three commercially available feline hydrolyzed dry diets before and after feeding exclusively for 6 weeks compared to a basal diet containing these proteins intact in a small number of individual healthy immunotolerant cats with known diet histories. Whole blood was used in the stimulation assays rather than peripheral blood mononuclear cells (PBMCs) in order to mimic the natural environment of the cells, to prevent phenotypic changes that may occur with isolation of cells, and to reduce the volume of blood needed. This technique has been shown to be comparable with the use of PBMCs [20]. Although whole blood stimulation assays have been performed using different stimulants [8, 9] in companion animals, to the authors’ knowledge, commercial pet foods have not been previously utilized. Therefore, this pilot study described for the first time, the cytokine response of feline whole blood when stimulated with different commercial diets.

This study demonstrated that the majority of cats produced detectable amounts of IL-10 when their whole blood was directly stimulated with the basal diet. This may suggest that at least 1 of the mechanisms for immunological tolerance to diet in cats may occur via the production of IL-10. However, further studies utilizing a negative control that contains protein that the cats have never been sensitized to, such as sterile human albumin would be needed to confirm that the IL-10 responses seen in our study is specific to the dietary antigens present in the tested diets and therefore may be representative of immunological tolerance. Interleukin-10 is an anti-inflammatory cytokine produced by both the innate and adaptive immune system and IL-10 is able to inhibit both Th-1 and Th-2 cytokines, as well as the expression of autoimmune and pro-inflammatory conditions [21]. One study in humans reported that natural tolerance to foods was associated with increased amounts of IL-10 producing peripheral blood mononuclear cells [22]. A second study proposed a role for serum IL-10 as a useful marker in the diagnosis of food tolerance in humans [23]. However, further studies will be needed to determine if IL-10 can be used as a marker for dietary immunotolerance in companion animals.

Interestingly, this study documented at least a 4-fold difference in individual IL-10 concentrations when ex-vivo whole blood was stimulated with the basal diet. This may be due to differences in peripheral lymphocyte concentrations between the cats. Unfortunately, the peripheral lymphocyte count was not determined and therefore the numbers per well for each cat could not be standardized. Therefore, future studies should focus on standardizing the number of lymphocytes per well for each cat as this may then allow for direct comparisons between individual cats to be made. In addition, healthy cats have serum antibodies to food proteins [24], therefore any antigen specific immunoglobulins in whole blood from the cats used in our study may have bound to dietary antigens, resulting in a lower or higher cytokine response depending on the characteristics of the antibody. This may have also resulted in the variation of IL-10 concentrations seen in our study. Therefore, future studies should also focus on the correlation of cytokine production with dietary antigen specific immunoglobulins to determine the effects of these antibodies on subsequent cytokine production.

Although this study documented that there was no significant difference in IL-10 concentrations following stimulation with the basal diet before and after 6 weeks of the hydrolyzed diet (Fig. 1), two of the seven cats had a lower IL-10 concentration after the 6 weeks compared to before. These temporal differences in IL-10 concentrations in individual cats may have been due to differences in lymphocyte numbers per well and dietary antigen specific immunoglobulins at the two time points. Therefore, future studies should account for the possibility of variation in lymphocyte counts and dietary antigen specific immunoglobulins when interpreting temporal differences in cytokine concentrations in individual cats.

Some of the immunotolerant cats in this study produced detectable IL-10 when stimulated with the hydrolyzed diets; one cat to the hydrolyzed chicken based diet and two cats to the hydrolyzed soy based diet. Two of these cats, one from each dietary group produced detectable IL-10 prior to consuming the hydrolyzed diet. All nine cats in this study had been sensitized to chicken and soy, as both ingredients were present intact in the previously fed diets, including the basal diet. This may suggest that if an animal was allergic to chicken or soy, then they may be able to recognize and react to the hydrolyzed forms of these proteins. This has been shown in one study, where 21% of dogs sensitized to the intact protein still reacted adversely to the hydrolyzed diet [6]. One possible cause for the persistence of clinical signs could be due to retention of larger sized or intact proteins in the hydrolyzed diet. Therefore, determining not just the average but also the range of sizes of hydrolyzed proteins in these diets and correlating this to the production of IL-10 in these cats may be beneficial to help predict tolerance.

None of the three cats in the hydroSC group produced detectable amounts of TNF-alpha, IL-10, or IL-4 when stimulated with their respective hydrolyzed soy and chicken based diet, prior to or after 6 weeks of consumption. It is possible that this hydrolyzed diet did not elicit a cytokine response due to the size and nature of the hydrolyzed proteins. However, according to the manufacturer of this diet, the average size of the dietary protein is 12 kDa, whereas the other two hydrolyzed diets are reported to have an average size of 10 kDa or less. However, the range of protein sizes for each diet was unspecified. If larger proteins persisted after hydrolysis, this might explain the discrepancy between the average protein size of the hydrolyzed diet and the respective cytokine response. Also, this study was conducted over a 6-week period and therefore it is possible that with a longer duration of consumption of the hydrolyzed soy and chicken based diet a cytokine response may have been seen. In addition, the individual differences in production of IL-10 to different hydrolyzed diets could also be explained by potential differences in peripheral lymphocyte count and the presence of dietary antigen specific immunoglobulins.

Interestingly, two of three of the cats in the hydroSC group produced TNF-alpha when stimulated with the basal diet following the 6-week diet trial. These were the only cats that produced no detectable IL-10 when their ex-vivo whole blood was stimulated with the basal diet following 6-weeks of hydrolyzed diet. The exact reason for this result is unknown, but the lack of IL-10 production may have prevented dampening of the TNF-alpha response that normally occurs following IL-10 production. In addition, although the dietary stimulants used in the ex-vivo whole blood stimulation assays had been filtered using a 0.22 μm filter, it is possible that bacterial remnants that are known to stimulate pattern recognition receptors (PRR) such as lipopolysaccharide (LPS) may have been present and could have resulted in the TNF-alpha response in these two cats. However, as the same batch of dietary extract was used for all nine cats and the majority of cats did not produce TNF-alpha on stimulation with these extracts, it is unlikely that LPS or other PRR ligands were present in significant amounts. However, specific measurement of PRR ligands should have been performed in order to refute this possibility.

The aim of this pilot study was to describe the immunological effects of three commercially available feline hydrolyzed dry diets before and after feeding exclusively for 6 weeks compared to a basal diet containing these proteins intact in individual healthy immunotolerant cats using ex-vivo whole blood stimulation assays. A small number of cats were enrolled in this pilot study in an effort to reduce the number of animals used in a research study with an unknown outcome. In addition, only healthy immunotolerant cats were used. Several studies have shown that various non-allergic animal models can be used to assess immunogenicity of human hydrolyzed milk or rice formulas [25–28]. In addition, one study in healthy cats demonstrated immunogenicity to dietary proteins when fed as either aqueous suspensions or as part of canned diets [24]. Therefore, future studies will focus on the use of this assay in a larger number of immunologically naïve, sensitized or clinically hypersensitized cats to determine if the cytokine response to hydrolyzed diets is similar to their basal diets containing the same proteins intact. This study focused on ex-vivo whole blood incubation for 24 h as this time frame was shown to result in measurable cytokine secretion in healthy immunotolerant children following whole blood stimulation with milk antigen, after which time lower levels were detected due to degradation [7]. Although our study was able to detect measurable amounts of IL-10 protein in the supernatants of the majority of cats following stimulation with their basal diet and in some cats following stimulation with some hydrolyzed commercial diets after 24 h, a longer incubation time may have been needed to fully assess lymphocyte derived cytokine production. Therefore, future studies will aim to focus on different incubation times to help confirm an optimum time for cytokine production following incubation with dietary antigens. In addition, future studies will also focus on the use of ex-vivo intestinal biopsy stimulation assays to determine the correlation of cytokine production between whole blood and intestinal mucosa to confirm if whole blood can be used as a surrogate for intestinal tissue when stimulated with diet.

## Conclusion

In conclusion, this pilot study for the first time showed that the majority of cats produced IL-10 to their basal diet using an ex-vivo whole blood stimulation assay. Similarly, some cats produced IL-10 to certain hydrolyzed therapeutic diets. This may suggest that in some healthy immunotolerant cats, some hydrolyzed diets may elicit a similar cytokine response compared to their basal diet, which contained the same proteins intact, however studies using a larger number of cats and addressing some of the limitations of the current study would be needed to corroborate this. In addition, further studies are needed to determine the immunological response to basal and hydrolyzed diets in immunologically naïve, sensitized or clinically hypersensitized cats.

## Abbreviations

AFR: Adverse food reaction
HydroC: Hydrolyzed chicken based diet
HydroS: Hydrolyzed soy based diet
HydroSC: Hydrolyzed soy and chicken based diet
IL: Interleukin
TNF: Tumor necrosis factor
RPMI: Roswell Park Memorial Institute
PBS: Phosphate buffered saline
PBMC: Peripheral blood mononuclear cells

## Footnotes

1 Whiskas® Kitten Dry with Chicken. Mars Incorporated, Fairfax County, VA. Main ingredients: poultry by-product meal, corn gluten meal, soybean meal, brewers rice, ground yellow corn, ground wheat, animal fat, and natural chicken and turkey flavors.

2 Purina Cat Chow® Complete Formula® dry cat food. Nestle Purina PetCare Company, St Louis, MO. Ingredients: poultry by-product meal, corn meal, corn gluten meal, ground whole wheat, brewers rice, soy flour, animal fat preserved with mixed-tocopherols, fish meal, animal liver flavor, and meat and bone meal.

3 Hill’s Prescription Diet Feline z/d dry food. Hill’s Pet Nutrition Inc., Topeka, KS. Ingredients: brewers rice, hydrolyzed chicken liver, soybean oil, powdered cellulose, and chicken liver flavor.

4 Purina Pro Plan Veterinary Diets Feline HA dry food. Ingredients: rice starch, hydrolyzed soy protein isolate, partially hydrogenated canola oil, hydrolyzed chicken liver, tricalcium phosphate, powdered cellulose, corn oil, and hydrolyzed chicken.

5 Royal Canin Veterinary Diet Feline Hydrolyzed Protein dry food. Royal Canin Inc. St. Charles, MO. Ingredients: brewers rice, hydrolyzed soy protein, and chicken fat.

6 50 ml Sterile Centrifuge Tubes. Corning®. Corning Incorporated, Corning, NY.

7 Millex ® GP Filter Unit, PES Membrane. Merck Millipore Ltd. Tullagreen, Co. Cork, IRL.

8 Sterile culture hood, Class II Type S/B3.

9 Bradford Reagent. Amresco, Solon, OH.

10 PBS. Hyclone Laboratories, South Logan, UT.

11 RPMI with L-glutamine, Corning Cellgro ®. Mediatech, Inc. Manassas, VA.

12 Corning Costar ®24 Well Plate, individually wrapped, sterile.

13 Hera Cell Incubator. Thermo Fischer Scientific Inc. Waltham, MA.

14 Feline TNF-alpha DuoSet ELISA. R&D Systems, Inc. Minneapolis, MN.

15 Feline IL-10 DuoSet ELISA. R&D Systems, Inc.

16 Feline IL-4 DuoSet ELISA. R&D Systems, Inc.

17 Bio-Tek Synergy H1 Multi-Mode Reader. Bio-Tek Instruments Inc. Winooski, VT.

18 Gen5 Microplate Reader and Imager Software. Bio-Tek Instruments Inc.
